# Editorial: Multi- and super-disciplinary approaches to plant Si and phytolith research

**DOI:** 10.3389/fpls.2026.1802031

**Published:** 2026-03-05

**Authors:** Ofir Katz, Mikhail S. Blinnikov, Mariana Fernández Honaine

**Affiliations:** 1 Dead Sea and Arava Science Center, Mt. Masada, Israel; 2 Ben-Gurion University of the Negev, Eilat, Israel; 3 Department of Atmospheric and Earth Sciences, St. Cloud State University, St. Cloud, MN, United States; 4 Instituto de Geología de Costas y del Cuaternario, Facultad de Ciencias Exactas/Universidad Nacional de Mar del Plata-CICPBA, Mar del Plata, Argentina; 5 Instituto de Investigaciones Marinas y Costeras, Facultad de Ciencias Exactas/Universidad Nacional de Mar del Plata-CONICET, Mar del Plata, Argentina

**Keywords:** archaeology, botany, phytolith, silica, silicon

German polymath Christian Gottfried Ehrenberg (1795–1876) was the one who coined the word phytolith to describe microscopic mineral bodies within plants. A portmanteau of Greek “phyton” (plant) and “lithos” (rock), the word encapsulates the multidisciplinary nature of these bodies and, in particular of plant silica, in between botany and geology. And indeed, plant silicon and phytoliths are strongly linked to many disciplines, crossing many disciplinary boundaries to a degree that oftentimes shatters such boundaries altogether (Katz, 2018). Botany, ecology, biogeography, pedology, palaeoclimatology and archaeology (amongst others)—plant silicon and phytoliths offer many opportunities for new discoveries and applications (Blinnikov and Yost, 2023).

Ehrenberg was also the person responsible for the first phytolith study in the Levant, more specifically where the Jordan river flows into the Dead Sea (Ehrenberg, 1849). On the shores of the same lake, in September 2023, the International Phytolith Society held its 13^th^ International Meeting on Phytolith Research, with talks and posters devoted to studies in multiple disciplines and to studies that cross or break the boundaries among disciplines. Frontiers in Plant Science was generous enough to support this meeting, leading to the compilation of this Research Topic. In line with the multidisciplinary and super disciplinary nature of plant silicon and phytolith research, this Research Topic covers many disciplines and various Frontiers journals (the assignment of manuscripts to journals was therefore not always straightforward).

Plant silicon and phytoliths are relevant for both the past and the future. Phytoliths’ durability, morphological versatility and large quantities make them a good environmental proxy. In ecological/landscape contexts, they provide valuable information about climate and land use changes. In archaeological contexts, they inform us of past plant utilization and human adaptations to local environments. In both cases, the implications for creating a sustainable future are clear.

Two contributions are archaeological by nature. Ferrara et al. present a method for extracting phytoliths from pottery powder used in organic residue analysis, which is a promising tool for studying the utilization of plants from one of the best contexts possible—the very vessels in which they were stored. Poliakova et al. studied palm phytoliths from palm-leaf manuscripts, with great potential applications for studying both the provenance of the manuscripts and local paleoecological reconstructions. Extending this method to other materials, such as papyrus, shows a great promise.

Two more contributions link the past with the future. Hermans et al. present new computational approach to studying and classifying ELONGATE DENDRITIC phytoliths as a means of distinguishing between wild and domesticated cereal variants, with clear application in tracking cereal domestication in archaeology. Ferrara et al. demonstrate the power of using phytoliths in tracing landscape history by analyzing phytoliths from soil profiles in olive agroecosystems. Such studies can deliver valuable knowledge for understanding land use changes.

Lastly, Kurze et al. look at extant ecology, studying the variations in silicon concentrations within a grass species along an aridity gradient. Unravelling how natural selection has shaped such variations is helpful for understanding how plant silica contributes to plant adaptation to climate change. With evolution and climate change, past and future are both present in this contribution as well.

These contributions have shown the diverse applications that plant silica and phytoliths studies have on multiple disciplines and, also, the interaction about them.

Finally, we wish to extend our gratitude to the contributing authors and to the reviewers.
